# Prenatal Exposure to Bisphenol A and/or Diethylhexyl Phthalate Impacts Brain Monoamine Levels in Rat Offspring

**DOI:** 10.3390/jox14030058

**Published:** 2024-08-01

**Authors:** Amrita Kaimal, Jessica M. Hooversmith, Maryam H. Al Mansi, Philip V. Holmes, Puliyur S. MohanKumar, Sheba M. J. MohanKumar

**Affiliations:** 1 Biomedical and Translational Sciences Institute, Neuroscience Division, University of Georgia, Athens, GA 30602, USA; 2 Department of Biomedical Sciences, College of Veterinary Medicine, University of Georgia, Athens, GA 30602, USA

**Keywords:** bisphenol A (BPA), diethylhexyl phthalate (DEHP), prenatal, monoamine neurotransmitters, sex differences, endocrine-disrupting chemical (EDC) mixtures

## Abstract

This study examines the sex-specific effects of gestational exposure (days 6–21) to endocrine-disrupting chemicals such as bisphenol A (BPA), diethylhexyl phthalate (DEHP), or their combination on brain monoamine levels that play an important role in regulating behavior. Pregnant Sprague–Dawley rats were orally administered saline, low doses (5 µg/kg BW/day) of BPA or DEHP, and their combination or a high dose (7.5 mg/kg BW/day) of DEHP alone or in combination with BPA during pregnancy. The offspring were subjected to a behavioral test and sacrificed in adulthood, and the brains were analyzed for neurotransmitter levels. In the paraventricular nucleus, there was a marked reduction in dopamine levels (*p* < 0.01) in male offspring from the BPA, DEHP, and B + D (HD) groups, which correlated well with their shock probe defensive burying times. Neurotransmitter changes in all brain regions examined were significant in female offspring, with DEHP (HD) females being affected the most, followed by the B + D groups. BPA and/or DEHP (LD) increased monoamine turnover in a region-specific manner in male offspring (*p* < 0.05). Overall, prenatal exposure to BPA, DEHP, or their combination alters monoamine levels in a brain region-specific, sex-specific, and dose-dependent manner, which could have implications for their behavioral and neuroendocrine effects.

## 1. Introduction

Chemicals such as bisphenol A (BPA) and diethylhexyl phthalate (DEHP) have a ubiquitous presence in the environment due to the widespread use of plastics. More recently, they have been detected globally in groundwater sources through plastic contamination of landfills and sludge from sewage and wastewater [1]. Exposure to these endocrine-disruption chemicals (EDCs) has been correlated with a variety of neurobehavioral disorders, including mood disorders, attention deficit hyperactivity disorder (ADHD), and autism [2,3]. An interesting feature about these disorders is the presence of a sex bias in their prevalence, with females showing higher rates of anxiety and depression, whereas an increased proportion of males have ADHD and autism [4,5,6]. EDC exposure during the in utero period is especially concerning, as these chemicals can easily cross the placental barrier in pregnant women [7,8], which can interfere with the development of sexually dimorphic systems and produce irreversible effects on fetal neurodevelopment [9,10,11]. Additionally, it is highly likely that EDCs such as BPA and DEHP may be present as mixtures in the environment or in combination with one another. The effects of EDC mixtures are known to be more complex and differ from those of individual EDCs [12,13]. Currently, the extent to which EDC mixtures affect neurobiological mechanisms following prenatal exposure is unknown. 

Monoamines, including norepinephrine (NE), dopamine (DA), and serotonin (5-HT), have long been implicated in the development and progression of anxiety and mood disorders [14,15,16], but the impact of prenatal BPA and/or DEHP exposure on monoaminergic activity in the brain is unclear. Perinatal exposure to BPA can dose- and sex-dependently modify levels of NE, 5-HT, and its metabolite 5-hydroxyindoleacetic acid (5-HIAA), as well as DA and its metabolite 3,4-dihydroxyphenylacetic acid (DOPAC) in brain areas including the forebrain and hypothalamus in male and female rodents [17,18,19]. Specifically, BPA exposure can interfere with DA and 5-HT synthesis in the prefrontal cortex (PFC) [20] and reduce 5-HT levels in the hippocampus [21] of adult male offspring. Six-month-old adult female offspring show decreased DOPAC/DA ratio, or dopamine turnover, in the brain following perinatal treatment with BPA doses as low as 10 µg/kg [22]. Additionally, perinatal BPA exposure can disrupt sexual dimorphism of the noradrenergic system [23,24].

On the other hand, much less is known about the repercussions of DEHP alone and BPA + DEHP combinations on neurotransmitter systems. Gestational DEHP at both low (20 µg/kg) and high doses (750 mg/kg) have been found to transgenerationally affect DA receptor expression levels in the amygdala in mice offspring [25]. However, the potential changes to other neurotransmitter systems in offspring with in utero low-dose DEHP exposure are largely unexplored. A recent study discovered reductions in DA and 5-HT in male offspring following perinatal exposures to high-dose BPA (50 mg/kg) or DEHP (30 mg/kg), both independently and in combination with one another [26]. This was coupled with alterations in the reuptake and biotransformation mechanisms of these neurotransmitters. It will be beneficial to further determine the ways in which monoaminergic activity is affected following prenatal exposure to low doses of these individual EDCs, as well as their mixtures, to obtain a clearer picture of the impact of EDC exposure on the brain. 

The purpose of this study was to create a profile of brain neurotransmitter and metabolite concentrations in adult offspring that were exposed to BPA, low-dose DEHP, or high-dose DEHP either individually or in combination during gestation. This study is a continuation of a recently published study from our lab focusing on the behavioral effects of these exposures [27]. We hypothesize that prenatal EDC exposure, particularly the mixture of BPA + DEHP, would lead to sex-specific and dose-related alterations in monoaminergic activity in specific brain regions that regulate behavior. 

## 2. Materials and Methods

### 2.1. Animals

Adult female Sprague–Dawley rats (Envigo, Indianapolis, IN, USA) were housed in light- and temperature-controlled rooms, and food and water were provided ad libitum as previously described [27]. Female breeders were randomly assigned one male rat when they were in proestrus and were bred for one day. The day of copulation represented gestational day 0 (GD0). The National Institutes of Health’s *Guide for the Care and Use of Laboratory Animals* was used to develop experimental protocols, and these were approved by the Institutional Animal Care and Use Committee (IACUC) at the University of Georgia. 

### 2.2. Chemicals

BPA (Lot MKBH2096V; Catalog No. 239658; Purity: ≥99.0%) and DEHP (Lot BCBR8079V; Catalog No. 36735; Purity: ≥98.0%) were purchased from Sigma Aldrich (St. Louis, MO, USA). Dimethylsulfoxide (DMSO) was used to prepare stock solutions to ensure complete dissolution of the EDCs and to facilitate reconstitution in saline so as to avoid irritation caused by DMSO. BPA and DEHP were dissolved at 1 μg/μL, and high-dose DEHP at 1 mg/μL. Doses for oral administration were calculated daily based on BW and mixed with 20 μL Phosphate Buffered Saline (PBS). Animals received the oral dose of EDC or EDC mixture every day from GD 6-21 as per Table 1 below. A micropipette was used to discharge the dose quickly with minimal stress to the pregnant dam. 

Only a single low dose of BPA was used as a positive control. The dose of BPA was selected based on the fact that it is considerably lower than the Environmental Protection Agency (EPA) established no-observed-adverse-effect-level (NOAEL) [28], as well as tolerable daily intake (TDI) doses [29]. Moreover, the dose used in the study is comparable to estimated human exposures (0.4–5 μg/kg/day) [30]. The high dose of DEHP used in our study is higher than the established NOAEL dose of 4.8 mg/kg/day [31], whereas the low DEHP dose is significantly lower than this. Additionally, the lower dose of DEHP used is within the range of the TDI of DEHP in humans (0.5–25 μg/kg/day) [32] and is below the EPA reference dose as described previously [27,33].

### 2.3. Experimental Design

**Figure 1:**
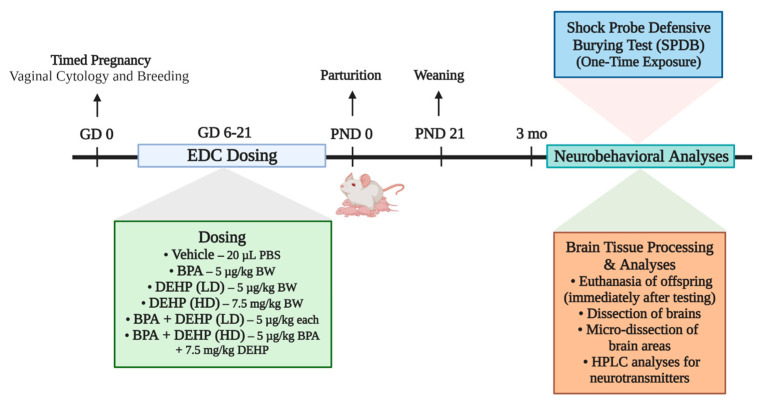
Schematic of the experimental design used for the study. Pregnant Sprague–Dawley dams were administered vehicle (*n* = 7), BPA (*n* = 7), low-dose (LD) DEHP (*n* = 6), high-dose (HD) DEHP (*n* = 6), a mixture of BPA + LD-DEHP (*n* = 6), or a mixture of BPA + HD-DEHP (*n* = 7) during days 6–21 of pregnancy. Adult offspring were subjected to the Shock Probe Defensive Burying test (SPDB) and immediately euthanized. The medial prefrontal cortex (mPFC), paraventricular nucleus (PVN), basolateral amygdala (BLA), and hippocampus (HC) were isolated from the brains and analyzed for neurotransmitter levels using HPLC-EC. The schematic above was created using Biorender.com.

Figure 1 depicts the experimental design used for this study. The dam was the experimental unit, and a representative male and female offspring from each dam was used in the study. Pregnant dams were assigned to one of six different treatment groups provided below.

We used 6 male and 6 female offspring from each group (12 animals total per group) when they were 4–5 months old. The standard = RAND() function was used to obtain random assignment numbers in Microsoft Excel. Animals were housed in temperature-controlled animal rooms maintained at 23 ± 1 °C. Lighting schedules were reversed, and behavioral testing was carried out in the dark, as described previously [27]. Animals were provided rat chow and water *ad libitum*. After behavioral testing, the animals were sacrificed by rapid decapitation, and the brains were harvested and frozen for neurotransmitter analysis. 

### 2.4. Shock Probe Defensive Burying Test (SPDB)

This behavioral test was conducted as described in Kaimal et al.2023 [27]. Animals were administered a mild shock of 3 mA DC described before [34]. Behavioral testing occurred during the dark cycle, and animals were tested under red lights. The response of the animal was recorded with a direct overhead webcam (Microsoft, Redmond, WA, USA). The animal generally responds to the insult by burying the probe. The amount of time the animals spent burying the probe was measured during the 10 min testing session, and all videos were manually scored by the double-blind method. This test is very effective in elucidating defensive behavior in animals and can help to dissect coping strategies used by the subjects.

### 2.5. Tissue Collection and Preparation 

At the time of euthanasia, we ensured that all female offspring were in the same state of the estrous cycle (estrous) using vaginal cytology. A vaginal smear that contained predominantly sheets of cornified epithelial cells indicates that the animal is in estrous. Both males and females were euthanized by rapid decapitation immediately after behavioral testing was completed. The brains were harvested and frozen for sectioning and neurotransmitter analysis.

### 2.6. Brain Sectioning, Microdissection, and Neurotransmitter Analysis

The frozen brains were sectioned using a cryostat at 300 μm. The medial prefrontal cortex (mPFC), paraventricular nucleus of the hypothalamus (PVN), basolateral amygdala (BLA), and ventral subdivision of the hippocampus (HC) were micro-dissected on a cold stage using the Palkovits’ microdissection procedure with a stereotaxic brain atlas for reference [35]. Tissues were stored at −80 °C until neurotransmitter analysis. 

### 2.7. Neurotransmitter Analysis by HPLC-EC

HPLC-EC was used to analyze brain punches for neurotransmitter concentrations as previously described [36]. Brain punches were briefly homogenized in 0.05 M perchloric acid, and an aliquot was used for protein estimation using the MicroBCA assay. The remaining homogenate was centrifuged and used for HPLC analysis. The HPLC-EC system has been described before [36] and comprised of a pump (LC-20AD), autoinjector (SIL-20AD), column oven (CTO-20AD) with a C-18 reverse phase column (Phenomenex, Torrance, CA, USA) maintained at 37 °C (Shimadzu, Columbia, MD, USA). Electrochemical detection was accomplished using a dual glassy carbon electrode and an LC-4C detector (Bioanalytical Systems, West Lafayette, IN, USA). After neurotransmitter concentrations were obtained by analyzing the chromatograms, they were divided by the corresponding protein values that were measured in duplicate using a BCA protein assay kit, and neurotransmitter concentrations in tissue samples were expressed as pg/μg of protein. Besides actual neurotransmitter values, turnover rates for DA and 5-HT were obtained by dividing the concentrations of the metabolites by the concentration of the parent neurotransmitter.

### 2.8. Statistical Analysis

Statistical analysis was performed using GraphPad prism software (version 9.1.1). Differences in neurotransmitter levels were analyzed by two-way ANOVA with EDC exposure and sex as variables, followed by Tukey’s multiple comparisons post hoc test. DA levels in the PVN were analyzed by one-way ANOVA with EDC exposure as the dependent variable, followed by Fisher’s LSD test. In addition, a simple linear regression was performed to determine any correlations between PVN DA levels and SPDB burying times in male offspring. Statistical significance was indicated by a *p*-value < 0.05. 

## 3. Results

### 3.1. Medial Prefrontal Cortex

**Figure 2:**
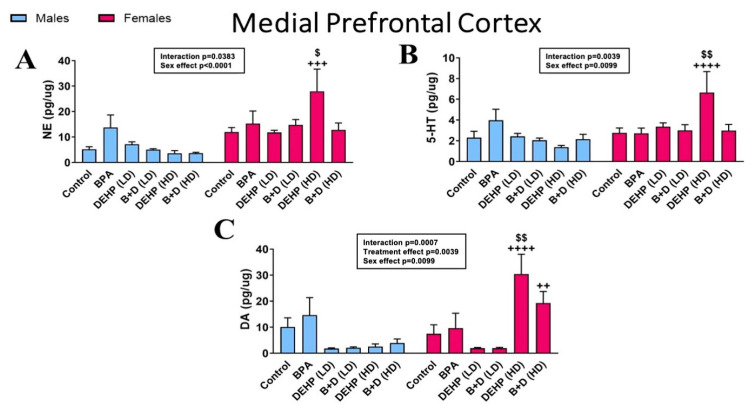
Impact of prenatal exposure to EDCs and their mixtures on monoamine levels in the medial prefrontal cortex (mPFC) of male and female offspring. (**A**) Norepinephrine (NE), (**B**) serotonin (5-HT), and (**C**) dopamine (DA) concentrations (mean ± SEM; pg/μg protein) in the mPFC are depicted in the figure. Doses of EDCs used are provided in the methods. ^$^ *p* < 0.05 and ^$$^ *p* < 0.01 indicate differences between control and EDC-exposed female offspring. ^++^ *p* < 0.01, ^+++^ *p* < 0.001, and ^++++^ *p* < 0.0001 indicate sex differences within the same treatment group.

In the mPFC (mean ± SEM; pg/μg protein), DEHP (HD) female offspring demonstrated robust increases in NE (27.9 ± 8.7; *p* = 0.0210) (Figure 2A), 5-HT (6.6 ± 2.0; *p* = 0.0080) (Figure 2B), and DA (30.4 ± 7.6; *p* = 0.0020) (Figure 2C) compared to control females. Sex differences were also identified in all three monoamines between DEHP (HD) males and females (*p* < 0.0001) and between B + D (HD) males and females in DA only (*p* = 0.0093). 

**Figure 3:**
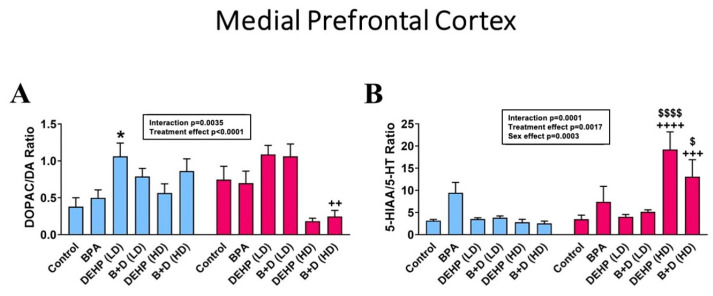
Monoamine turnover ratios in the medial prefrontal cortex (mPFC) of male and female rat offspring exposed prenatally to EDCs or their mixtures. (**A**) DOPAC/DA and (**B**) 5-HIAA/5-HT ratios (mean ± SEM) in the mPFC are shown in the figure. Data from adult males are represented by blue bars, and from females are represented by red bars. * indicates significant differences (*p* < 0.05) in male offspring. ^$^ *p* < 0.05 and ^$$$$^ *p* < 0.0001 indicate significant differences in female offspring. ^++^ *p* < 0.01, ^+++^ *p* < 0.001, and ^++++^ *p* < 0.0001 indicate sex differences within the same treatment group.

In terms of DOPAC/DA ratios (mean ± SEM), DEHP (LD) male offspring had significantly higher DA turnover (1.1 ± 0.2) compared to control male offspring (0.4 ± 0.1; *p* = 0.0117; Figure 3A). Additionally, B + D (HD) male offspring had increased DA turnover relative to their female counterparts (*p* = 0.0026). Serotonin turnover, as depicted by the 5-HIAA/5-HT ratio, was markedly increased in the mPFC in HD female offspring (Figure 3B). Female offspring in the DEHP (HD) (19.2 ± 4.0; *p* < 0.0001) and B + D (HD) (13.1 ± 3.8; *p* = 0.0140) groups had significantly higher serotonin turnover rates compared to control females (3.5 ± 0.9) and also their male counterparts (DEHP (HD): *p* < 0.0001; B + D (HD): *p* = 0.0004) (Figure 3B and Table 2).

Minor changes were discovered in DOPAC levels within the mPFC, which are displayed in Table 2. Sex differences were observed in the BPA and DEHP (HD) groups, with the two groups showing opposite differences between males and females. In 5-HIAA levels, DEHP (HD) female offspring once again had higher levels than control female offspring (*p* < 0.0001). Finally, females in both HD groups showed significant elevations in 5-HIAA metabolite levels compared to their male counterparts.

### 3.2. Paraventricular Nucleus of the Hypothalamus

**Figure 4:**
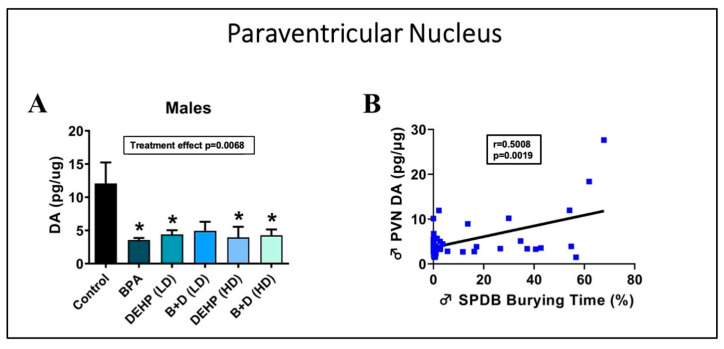
Changes in dopamine (DA) levels in the paraventricular nucleus (PVN) (**A**) and correlation between DA levels and SPDB burying time (**B**) in male offspring. * indicates significant differences (*p* < 0.05) between control and EDC-exposed male offspring.

Changes in DA levels (mean ± SEM; pg/μg protein) were especially remarkable in the PVN (Figure 4A). One-way ANOVA revealed that male offspring in the BPA (3.6 ± 0.3; *p* = 0.0093), DEHP (LD) (4.4 ± 0.6; *p* = 0.0323), DEHP (HD) (4.0 ± 1.6; *p* = 0.0202), and B + D (HD) (4.3 ± 0.9; *p* = 0.0205) groups had significantly lower DA levels than controls (12.1 ± 3.2). Furthermore, a simple linear regression indicated a significant moderate positive correlation (R = 0.5008, *p* = 0.0019) between DA levels in the PVN and the amount of time male rats spent burying the probe in the SPDB [26] (Figure 4B). This suggests a correlation between this behavior and DA levels in the PVN in male rats. Female offspring, on the other hand, did not demonstrate a significant change in burying behavior in the SPDB [26]. However, two-way ANOVA revealed that high-dose DEHP (*p* = 0.0528) or B + D (*p* = 0.0033) treatments increased DA levels in the PVN in female offspring compared to the corresponding controls (Table 2). These results clearly demonstrate sex-specific correlations between DA levels in the PVN and SPDB.

**Figure 5:**
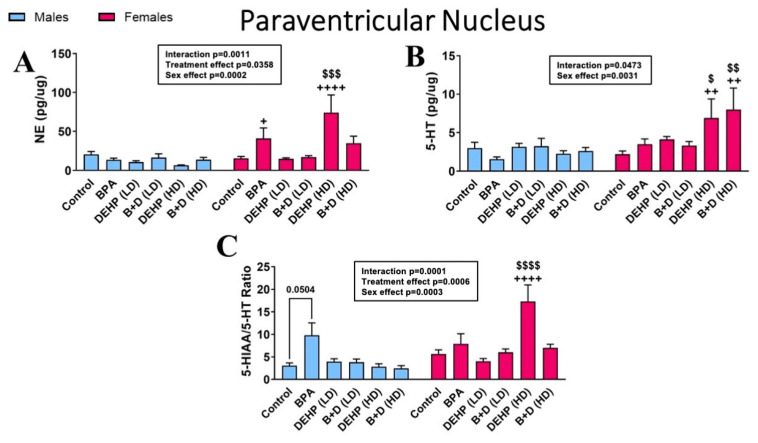
Impact of prenatal EDC exposure on monoamine levels and monoamine turnover ratios in the paraventricular nucleus (PVN) of rat offspring. (**A**) Norepinephrine (NE) levels (mean ± SEM; pg/μg protein), (**B**) serotonin (5-HT) levels (mean ± SEM; pg/μg protein), and (**C**) 5-HIAA/5-HT ratios (mean ± SEM) in the PVN are shown). ^$^ *p* < 0.05, ^$$^ *p* < 0.01, ^$$$^ *p* < 0.001, and ^$$$$^ *p* < 0.0001 indicate significant differences caused by treatment in female offspring. ^+^ *p* < 0.05, ^++^ *p* < 0.01, and ^++++^ *p* < 0.0001 indicate significant differences between the two sexes within the same treatment group.

Various EDC- and/or sex-dependent effects were observed in the other monoamines, metabolites, and turnover ratios in the PVN (Figure 5 and Table 2). DEHP (HD) female offspring, in particular, exhibited robust hyperactivity in levels of NE (74.1 ± 22.7 vs. 15.4 ± 2.5; *p* = 0.0001) (Figure 5A), 5-HT (6.9 ± 2.5 vs. 2.2 ± 0.4; *p* = 0.0490) (Figure 5B), DOPAC (6.0 ± 2.2 vs. 1.4 ± 0.4; *p* = 0.0009), 5-HIAA (103.6 ± 42.4 vs. 11.0 ± 1.8; *p* = 0.0003), and 5-HT turnover (17.3 ± 3.7 vs. 5.7 ± 0.9; *p* < 0.0001) compared to their control counterparts. B + D (HD) females also appeared to mimic this response to a greater extent, but in 5-HT (8.0 ± 2.8 vs. 2.2 ± 0.4; *p* = 0.0072) and DA (4.3 ± 0.9 vs. 12.1 ± 3.2; *p* = 0.0033) levels only. Finally, BPA male offspring (*p* = 0.0504) demonstrated increased 5-HIAA/5-HT ratios compared to their control counterparts (Figure 5C). 

### 3.3. Basolateral Amygdala

**Figure 6:**
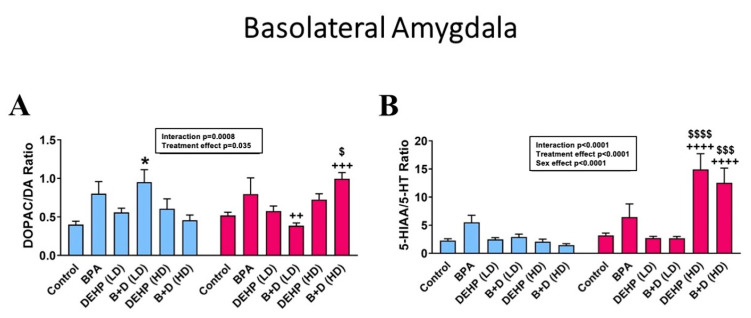
Changes in monoamine turnover ratios in the basolateral amygdala (BLA) of male and female rat offspring prenatally exposed to EDCs or their mixtures. DOPAC/DA is depicted in panel (**A**), and 5-HIAA/5-HT ratios are depicted in panel (**B**). * indicates significant differences due to EDC treatment in male offspring. ^$^ *p* < 0.05, ^$$$^ *p* < 0.001, and ^$$$$^ *p* < 0.0001 indicate significant differences due to treatment in female offspring. ^++^ *p* < 0.01, ^+++^ *p* < 0.001, and ^++++^ *p* < 0.0001 indicate sex differences within the same treatment group.

Interestingly, no effects of EDC exposure were observed in monoamines in the BLA (Table 2). However, strong sex differences were uncovered in this region, particularly in NE and DA levels. A majority of the EDC groups displayed sex differences in BLA DA concentrations. DEHP (LD) male and female offspring were an exception, with very similar DA levels to one another, suggestive of an abolishment of sex differences. EDC exposure appeared to impact monoamine turnover rates (mean ± SEM) in the BLA instead (Figure 6). In particular, male offspring with low-dose B + D treatment (1.0 ± 0.2 vs. 0.4 ± 0.0; *p* = 0.0137), whereas female offspring with the corresponding high-dose treatment (1.0 ± 0.1 vs. 0.5 ± 0.0; *p* = 0.0323) had significantly higher DOPAC/DA ratios than their control counterparts, implying a sex- and dose-dependent effect. Sex differences were also generated in these two groups, with both groups showing a reversal of sex differences compared to each other. Finally, metabolite levels revealed that, once again, high-dose DEHP and B + D female offspring had elevated levels of both DOPAC and 5-HIAA compared to their male counterparts and control females (Table 2).

### 3.4. Hippocampus

**Figure 7:**
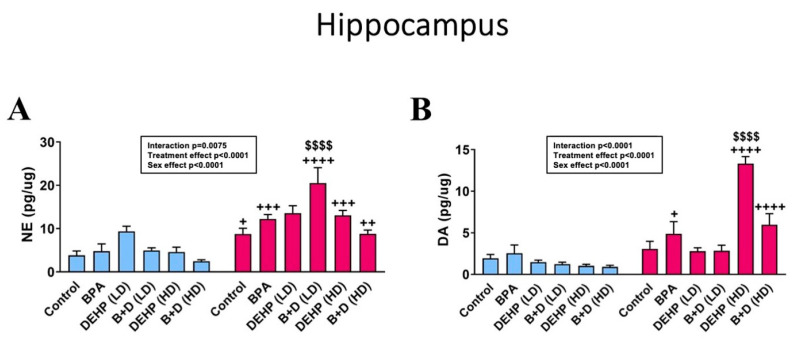
Monoamine levels in the hippocampus (HC) of female and male rat offspring that were prenatally exposed to EDCs or their mixtures. (**A**) Norepinephrine (NE) and (**B**) dopamine (DA) concentrations (mean ± SEM; pg/μg protein) in the HC are depicted in the figure. ^$$$$^ *p* < 0.0001 indicates significant differences between control and EDC-exposed female offspring. ^+^ *p* < 0.05, ^++^ *p* < 0.01, ^+++^ *p* < 0.001, and ^++++^ *p* < 0.0001 indicate significant sex differences within the same treatment group.

We found a pronounced increase in hippocampal NE levels (mean ± SEM; pg/μg protein) (Figure 7A) in B + D (LD) female offspring in comparison with control females (20.5 ± 3.6 vs. 8.8 ± 1.3; *p* < 0.0001). Furthermore, sex differences were observed in NE levels in all groups, but these were abolished in DEHP (LD) offspring again. DA levels in the HC were, not surprisingly, substantially increased in DEHP (HD) female offspring compared to their control counterparts (13.3 ± 0.9 vs. 3.1 ± 0.9; *p* < 0.0001). Apart from that, some minor sex differences were observed in 5-HT, metabolites, and DOPAC/DA ratio, which are detailed in Table 2. 

## 4. Discussion

The objective of the present study was to determine the effects on brain NE, DA, 5-HT, DOPAC, and 5-HIAA levels following prenatal treatment with various doses of EDCs, either individually or in combination. One of the major findings of the present study was the reduction in DA concentrations in the PVN within male offspring from all EDC groups except for B + D (LD). Especially fascinating was the fact that this appeared to parallel the reduced burying time we previously noted in the shock probe defensive burying test (SPDB) in male offspring from the very same EDC groups [27]. When we investigated this further, a significant moderate positive correlation was identified between SPDB burying time and DA levels in the PVN of male offspring, suggesting that reduced burying is moderately correlated with lower levels of PVN DA. 

Reduced burying in the SPDB is often interpreted as a maladaptive decrease in active coping abilities [34]. Our findings imply that diminished PVN DA may underlie the reduced active coping defense strategies we found in males prenatally exposed to EDCs. Alterations in brain DA concentrations are commonly linked with a variety of stress-related psychopathologies, including major depressive disorder [37], posttraumatic stress disorder [38], and substance use disorder [39]. Furthermore, studies have established that an increase in brain dopaminergic activity occurs in response to acute physiological and psychological stressors [40]. In contrast, our study uncovered reduced DA concentrations in the male PVN, indicative of aberrant responses within the brain as a result of prenatal EDC treatment. Our findings imply potential dysfunctions within the mesocortical and/or mesolimbic dopaminergic pathways and may represent deficits in motivation or cognition instead. This particular outcome requires further investigation. Nevertheless, the lower PVN DA levels accompanied by deficits in active coping appear to be a sex-specific EDC mechanism of action that has not been identified before. 

A total of 5 μg/kg of DEHP exposure in our study led to increased dopamine turnover (DOPAC/DA ratio) in the mPFC of male offspring, along with lower PVN DA. Our prior experiments found that these DEHP (LD) male offspring exhibited heightened immobility in the SPDB [27]. Consistent with our study, acute stress is associated with increases in DOPAC/DA ratio in the mPFC as well as stress-induced immobility responses in male rats [41,42]. Furthermore, infusions of corticotropin-releasing factors can also increase PFC ratios of DOPAC/DA in male mice [43]. The results from our experiments support and expand these findings by showing that prenatal DEHP exposure at a low dose can exacerbate these effects in male offspring. 

Since DOPAC is formed from the catalysis of DA via monoamine oxidase (MAO) [44], it is reasonable to speculate that changes in MAO activity may underlie the elevated DOPAC/DA ratio we discovered in DEHP (LD) males. However, a very recent study discovered no changes in MAO activity in male offspring with perinatal high-dose DEHP treatment (30 mg/kg) [26] but a reduction in dopamine transporter levels instead. Future studies should explore the effects of LD-DEHP on DA reuptake and metabolism mechanisms. Another potential mechanism of action of DEHP to consider is its impact on the mesocortical dopaminergic system. The PFC is part of this system and receives dopaminergic innervation from the ventral tegmental area (VTA) [45]. Partial loss of mPFC DA during early postnatal development via electrolytic lesions of the VTA or intraventricular infusions of the neurotoxic 6-hydroxydopamine is linked to increased DA turnover in the mPFC of adult rats [46,47,48]. Therefore, low-dose DEHP exposure during the prenatal period may directly or indirectly lead to the loss of DA neurons, particularly in the PFC of male offspring, resulting in behavioral and dopaminergic alterations in adulthood.

Male and female offspring with DEHP (HD) exposure showed vastly different treatment effects. The males only showed dopaminergic reductions in the PVN, whereas the females had substantially greater concentrations of neurotransmitters, metabolites, or turnover rates in all brain regions examined. The results pertaining to monoaminergic activity in DEHP (HD) female offspring are especially fascinating because these offspring did not previously demonstrate any robust alterations in behavior [27]. Therefore, enhanced brain monoamine signaling and a lack of behavioral changes may suggest a neuroprotective effect of this dose of DEHP in female offspring. Consistent with this theory, studies have demonstrated that females show enhanced brain functioning, specifically in the HC, in response to postnatal DEHP treatment at 1–20 mg/kg, while their male counterparts display the opposite or no effects [49,50]. Hence, females may exhibit resilience following DEHP exposure, and our study further indicates that this effect is observable with prenatal exposure as well.

Treatment with a mix of BPA and DEHP increased DA turnover in the BLA in males treated with the low dose. Elevated amygdaloid DA turnover has been discovered in rats exposed to early life stress [51], as well as in rats with unilateral lesions of the mesocortical dopaminergic system, contributing to increased stress vulnerability [52]. Furthermore, perinatal LD BPA exposure can reduce whole brain DOPAC/DA ratio in adult female mice [22], whereas the effects of DEHP on the dopaminergic system are understudied. Our results reveal that prenatal treatment with a combination of BPA and DEHP (LD) in male rats does not alter behavior [27] but does affect DA neurotransmission, specifically in the BLA.

B + D (LD) female offspring, on the other hand, had increased hippocampal NE, whereas females with individual BPA or DEHP (LD) treatment showed no changes in monoaminergic activity. In terms of behavior, our B + D (LD) females engaged in increased immobility in the SPDB [27] and showed enhanced center zone exploration in the OFT. Immobilization stress has been demonstrated to enhance NE levels in the HC [53]. Moreover, NE is also released in the hippocampus following novelty exposure and arousal when the locus coeruleus–noradrenergic system is activated [54]. It is possible that hippocampal NE levels were elevated in response to novelty, which mediated the diverse behavioral effects observed.

In the B + D (HD) group, male offspring only demonstrated lower PVN DA, while female offspring showed higher concentrations of both DA and 5-HT in the PVN, as well as elevated turnover of these monoamines in the BLA and of 5-HT in the mPFC. However, these females did not show any robust changes in behavior based on our previous experiments despite their brain monoaminergic alterations [27]. Overall, B + D (HD) females displayed vast similarities with DEHP (HD) females in their neurobehavioral effects. This suggests that 7 mg/kg of DEHP individually and in combination with 5 μg/kg of BPA may have common mechanisms of action in female offspring. More precisely, when the two chemicals are combined at these respective doses, the effects of DEHP surpass those of BPA in female offspring, possibly as a result of the higher dose of DEHP canceling out the effects of the lower BPA dose.

The reason for neurotransmitter differences between the sexes is not clear; however, the role of sex hormones needs to be considered. It should be noted that the female rats were in a state of estrous at the time of sacrifice. During this state, female hormone levels are low and do not fluctuate [55], suggesting that how the brain is wired in both sexes possibly has a role to play in the neurotransmitter differences rather than sex hormones per se. Or it may indicate that prenatal exposures to EDCs alter the neurocircuitry at a very young age, producing sex-specific changes. Other possibilities include increased cytokine production that could promote a pro-oxidative stress environment in specific brain regions. Similar changes have been previously reported with prenatal BPA [56] and DEHP exposure [57]. 

The interaction of BPA and DEHP with estrogen receptors could be another contributing factor to the sex differences observed in this study. This is a highly complex phenomenon, and our understanding is confounded by the different types of estrogen receptors, their variable expression in the brain areas investigated in this study [58], and the differences in their expression in males and females [59]. While BPA binds weakly to the estrogen receptor [60], DEHP is capable of modulating them [61]. Besides the estrogen receptors, BPA and DEHP bind and affect CB1 [62,63], PAR alpha [64,65], and AhR [66,67] to various extents, resulting in numerous permutations and combinations of effects on the developing nervous system. These could lead to changes in synaptic architecture and function, which could potentially lead to alterations in neural circuits, as mentioned above.

## 5. Conclusions

To summarize, the findings of this study uncovered dose- and sex-dependent alterations in stress-regulating brain regions, including the mPFC, PVN, BLA, and HC. Male offspring from all treatment groups, with the exception of B + D (LD), had reduced DA in the PVN. In contrast, female offspring exposed to 7.5 mg/kg of DEHP alone and in combination with BPA had substantially enhanced monoaminergic activity, possibly validating a neuroprotective role of DEHP treatment in females. A total of 5 μg/kg of BPA and/or DEHP increased monoamine turnover in a brain region-specific manner in male offspring only. Finally, B + D exposure impacted offspring in a sex- and dose-dependent manner, with female offspring exposed to this mixture at the high dose being affected more and demonstrating similarities with DEHP (HD) females. Altogether, these findings fill in some of the gaps in EDC research, specifically in terms of the effects of EDC mixtures, neurobiological mechanisms, and sex differences. 

## Figures and Tables

**Table 1:** Animals included in each treatment group and the dose of EDC used for each group.

Treatment	Dose	Number of Animals Per Group
Control	20 μL Phosphate Buffered Saline (PBS)	7
BPA	5 μg/kg BW/day	7
DEHP low dose (LD)	5 μg/kg BW/day	6
DEHP high dose (HD)	7.5 mg/kg BW/day	6
BPA + LD-DEHP	5 μg/kg/day of BPA + 5 μg/kg/day of DEHP	6
BPA + HD-DEHP	5 μg/kg/day of BPA + 7.5 mg/kg/day of DEHP	7

**Table 2:** Neurotransmitter data of male and female offspring following low-dose (5 μg) and/or high-dose (7.5 mg) prenatal EDC exposure.

Neurotransmitter	Control		BPA (5 μg)		DEHP (5 μg)		BPA + 5 μg DEHP		DEHP (7.5 mg)		BPA + 7.5 mg DEHP		Sex Effect
	Male	Female	Male	Female	Male	Female	Male	Female	Male	Female	Male	Female	
**Medial Prefrontal Cortex**													
DOPAC (pg/μg)	2.0 ± 0.5	1.9 ± 0.2	4.7 ± 1.7 ^+^	2.3 ± 0.3 ^+^	1.6 ± 0.1	2.0 ± 0.2	1.7 ± 0.3	2.1 ± 0.7	1.2 ± 0.4 ^++^	4.5 ± 0.6 ^++^	2.4 ± 0.7	2.9 ± 0.5	NS
5-HIAA (pg/μg)	7.1 ± 1.8	8.0 ± 1.9	10.5 ± 2.2	32.0 ± 17.1	7.5 ± 0.6	11.3 ± 0.7	7.1 ± 0.9	13.8 ± 1.8	6.7 ± 3.0 ^++++^	109.2 ± 28.0 ^$$$$,++++^	4.1 ± 0.3 ^+^	37.1 ± 13.2 ^+^	*p* < 0.0001
**Paraventricular Nucleus**													
DA (pg/μg)	12.1 ± 3.2	5.8 ± 0.9	3.6 ± 0.3	12.7 ± 4.2	4.4 ± 0.6	7.2 ± 0.7	4.9 ± 1.4	6.2 ± 0.8	4.0 ± 1.6 ^++^	25.8 ± 5.9 ^++^	4.3 ± 0.9 ^++++^	31.2 ± 13.9 ^$$,++++^	*p* = 0.0008
DOPAC (pg/μg)	1.9 ± 0.2	1.4 ± 0.4	1.7 ± 0.5	2.9 ± 1.2	1.1 ± 0.1	1.3 ± 0.4	1.3 ± 0.2	1.1 ± 0.3	0.9 ± 0.1 ^++++^	6.0 ± 2.2 ^$$$,++++^	1.5 ± 0.2	1.7 ± 0.5	*p* = 0.0310
5-HIAA (pg/μg)	8.7 ± 1.5	11.0 ± 1.8	11.8 ± 1.8	24.4 ± 6.2	11.3 ± 0.8	15.9 ± 1.4	10.4 ± 1.6	18.2 ± 1.0	4.1 ± 0.3 ^++++^	103.6 ± 42.4 ^$$$,++++^	5.1 ± 0.4 ^++^	63.0 ± 30.2 ^++^	*p* = 0.0006
DOPAC/DA ratio	0.2 ± 0.1	0.2 ± 0.0	0.5 ± 0.1 ^+^	0.3 ± 0.1 ^+^	0.3 ± 0.1	0.2 ± 0.0	0.3 ± 0.0	0.2 ± 0.1	0.3 ± 0.1	0.2 ± 0.0	0.4 ± 0.1 ^++^	0.1 ± 0.0 ^++^	*p* = 0.0002
**Basolateral Amygdala**													
NE (pg/μg)	7.7 ± 2.2 ^+^	13.7 ± 1.8 ^+^	7.8 ± 1.8 ^++++^	22.0 ± 2.2 ^++++^	9.9 ± 0.5 ^+^	15.3 ± 1.2 ^+^	7.2 ± 0.7 ^+++^	17.6 ± 2.3 ^+++^	6.7 ± 1.3 ^++++^	20.6 ± 2.3 ^++++^	5.8 ± 1.0 ^+++^	14.7 ± 2.7 ^+++^	*p <* 0.0001
DA (pg/μg)	8.7 ± 1.7	12.4 ± 1.6	6.2 ± 1.3 ^+++^	16.2 ± 2.5^+++^	15.2 ± 2.5	15.1 ± 1.6	7.3 ± 1.0 ^++^	15.5 ± 1.6 ^++^	7.2 ± 1.9 ^+++^	18.5 ± 0.8 ^+++^	5.5 ± 1.4 ^++^	13.6 ± 3.3 ^++^	*p <* 0.0001
5-HT (pg/μg)	2.7 ± 0.4	3.9 ± 0.8	2.1 ± 0.4 ^++^	4.9 ± 1.0 ^++^	3.8 ± 0.4	5.0 ± 0.4	2.9 ± 0.6^+^	5.6 ± 0.9 ^+^	2.9 ± 0.7	3.5 ± 0.8	3.6 ± 0.6	3.0 ± 0.9	*p* = 0.0015
DOPAC (pg/μg)	3.5 ± 0.8	6.3 ± 0.7	4.2 ± 0.8 ^++^	10.9 ± 1.9 ^++^	8.2 ± 1.1	9.0 ± 1.7	7.0 ± 1.8	6.0 ± 0.8	3.8 ± 1.0 ^++++^	13.6 ± 1.7 ^$,++++^	2.2 ± 0.4 ^++++^	14.6 ± 4.1 ^$$,++++^	*p <* 0.0001
5-HIAA (pg/μg)	5.9 ± 1.2	10.8 ± 1.3	12.4 ± 3.7	24.7 ± 7.1	8.7 ± 0.5	13.0 ± 0.9	7.4 ± 0.6	14.0 ± 1.5	8.2 ± 3.2 ^++++^	43.8 ± 5.1 ^$$$,++++^	4.6 ± 0.6 ^++++^	39.3 ± 14.4 ^$$,++++^	*p <* 0.0001
**Hippocampus**													
5-HT (pg/μg)	0.9 ± 0.2 ^+^	2.0 ± 0.6 ^+^	1.8 ± 0.5	1.8 ± 0.3	1.6 ± 0.2	2.4 ± 0.3	1.0 ± 0.2 ^++^	2.7 ± 0.6 ^++^	0.9 ± 0.2	1.4 ± 0.2	0.9 ± 0.3	1.3 ± 0.2	*p* = 0.0009
DOPAC (pg/μg)	0.4 ± 0.1 ^+^	1.3 ± 0.4 ^+^	1.0 ± 0.5	0.9 ± 0.1	0.8 ± 0.2	1.1 ± 0.4	0.7 ± 0.1	0.7 ± 0.1	0.4 ± 0.1 ^++^	1.5 ± 0.5 ^++^	0.5 ± 0.2	1.1 ± 0.2	*p* = 0.0063
5-HIAA (pg/μg)	3.8 ± 0.8 ^+^	8.1 ± 2.6 ^+^	6.5 ± 1.5	9.5 ± 1.3	7.3 ± 0.5	10.0 ± 0.6	5.4 ± 0.8 ^++^	11.8 ± 2.1 ^++^	4.1 ± 1.1 ^+++^	13.0 ± 1.3 ^+++^	2.3 ± 0.2 ^+++^	10.7 ± 2.4 ^+++^	*p* < 0.0001
DOPAC/DA ratio	0.3 ± 0.1	0.5 ± 0.2	0.2 ± 0.1	0.3 ± 0.1	0.6 ± 0.1	0.4 ± 0.1	0.6 ± 0.1 ^+^	0.3 ± 0.1 ^+^	0.3 ± 0.1	0.1 ± 0.0	0.5 ± 0.1	0.3 ± 0.1	*p* = 0.0283
5-HIAA/5-HT ratio	4.2 ± 0.8	4.0 ± 1.0	8.0 ± 2.5	7.4 ± 2.3	4.9 ± 0.4	4.5 ± 0.6	5.4 ± 0.6	4.9 ± 0.8	4.9 ± 0.9	10.5 ± 2.6	3.4 ± 0.8	9.1 ± 1.5	NS

Note: EDC, endocrine-disrupting chemicals; BPA, bisphenol A; DEHP, di-(2-ethylhexyl) phthalate; NE, norepinephrine; DA, dopamine; 5-HT, serotonin; DOPAC, 3,4-dihydroxyphenylacetic acid; 5-HIAA, 5-hydroxyindoleacetic acid; NS, non-significant. Data are presented as mean ± SEM. ^$^ *p* < 0.05, ^$$^ *p* < 0.01, ^$$$^ *p* < 0.001, and ^$$$$^ *p* < 0.0001 indicate treatment differences in females. ^+^ *p* < 0.05, ^++^ *p* < 0.01, ^+++^ *p* < 0.001, and ^++++^ *p* < 0.0001 indicate sex differences between males within the same treatment group.

## Data Availability

Data generated during the course of these studies are available upon request.

## References

[1] Duenas-Moreno J., Mora A., Cervantes-Aviles P., Mahlknecht J. (2022). Groundwater contamination pathways of phthalates and bisphenol A: Origin, characteristics, transport, and fate—A review. Environ. Int..

[2] Kajta M., Wójtowicz A.K. (2013). Impact of endocrine-disrupting chemicals on neural development and the onset of neurological disorders. Pharmacol. Rep..

[3] Mustieles V., Pérez-Lobato R., Olea N., Fernández M.F. (2015). Bisphenol A: Human exposure and neurobehavior. Neurotoxicology.

[4] Altemus M., Sarvaiya N., Epperson C.N. (2014). Sex differences in anxiety and depression clinical perspectives. Front. Neuroendocrinol..

[5] Christensen D.L., Baio J., Van Naarden Braun K., Bilder D., Charles J., Constantino J.N., Daniels J., Durkin M.S., Fitzgerald R.T., Kurzius-Spencer M. (2016). Prevalence and Characteristics of Autism Spectrum Disorder Among Children Aged 8 Years—Autism and Developmental Disabilities Monitoring Network, 11 Sites, United States, 2012. MMWR Surveill Summ..

[6] Ramtekkar U.P., Reiersen A.M., Todorov A.A., Todd R.D. (2010). Sex and age differences in attention-deficit/hyperactivity disorder symptoms and diagnoses: Implications for DSM-V and ICD-11. J. Am. Acad. Child Adolesc. Psychiatry.

[7] Balakrishnan B., Henare K., Thorstensen E.B., Ponnampalam A.P., Mitchell M.D. (2010). Transfer of bisphenol A across the human placenta. Am. J. Obstet. Gynecol..

[8] Singh A.R., Lawrence W.H., Autian J. (1975). Maternal-fetal transfer of 14C-di-2-ethylhexyl phthalate and 14C-diethyl phthalate in rats. J. Pharm. Sci..

[9] Braun J.M. (2017). Early-life exposure to EDCs: Role in childhood obesity and neurodevelopment. Nat. Rev. Endocrinol..

[10] Nesan D., Kurrasch D.M. (2020). Gestational Exposure to Common Endocrine Disrupting Chemicals and Their Impact on Neurodevelopment and Behavior. Annu. Rev. Physiol..

[11] Rebuli M.E., Patisaul H.B. (2016). Assessment of sex specific endocrine disrupting effects in the prenatal and pre-pubertal rodent brain. J. Steroid Biochem. Mol. Biol..

[12] Biemann R., Fischer B., Santos A.N. (2014). Adipogenic effects of a combination of the endocrine-disrupting compounds bisphenol A, diethylhexylphthalate, and tributyltin. Obes. Facts..

[13] Suteau V., Briet C., Lebeault M., Gourdin L., Henrion D., Rodien P., Munier M. (2020). Human amniotic fluid-based exposure levels of phthalates and bisphenol A mixture reduce INSL3/RXFP2 signaling. Environ. Int..

[14] Hamon M., Blier P. (2013). Monoamine neurocircuitry in depression and strategies for new treatments. Prog. Neuropsychopharmacol. Biol. Psychiatry.

[15] Liu Y., Zhao J., Guo W. (2018). Emotional Roles of Mono-Aminergic Neurotransmitters in Major Depressive Disorder and Anxiety Disorders. Front. Psychol..

[16] Ruhé H.G., Mason N.S., Schene A.H. (2007). Mood is indirectly related to serotonin, norepinephrine and dopamine levels in humans: A meta-analysis of monoamine depletion studies. Mol. Psychiatry.

[17] Honma T., Miyagawa M., Suda M., Wang R.S., Kobayashi K., Sekiguchi S. (2006). Effects of perinatal exposure to bisphenol A on brain neurotransmitters in female rat offspring. Ind. Health.

[18] Nakamura K., Itoh K., Yoshimoto K., Sugimoto T., Fushiki S. (2010). Prenatal and lactational exposure to low-doses of bisphenol A alters brain monoamine concentration in adult mice. Neurosci Lett..

[19] Ogi H., Itoh K., Ikegaya H., Fushiki S. (2015). Alterations of neurotransmitter norepinephrine and gamma-aminobutyric acid correlate with murine behavioral perturbations related to bisphenol A exposure. Brain Dev..

[20] Castro B., Sánchez P., Miranda M.T., Torres J.M., Ortega E. (2015). Identification of dopamine- and serotonin-related genes modulated by bisphenol A in the prefrontal cortex of male rats. Chemosphere.

[21] Xin F., Fischer E., Krapp C., Krizman E.N., Lan Y., Mesaros C., Snyder N.W., Bansal A., Robinson M.B., Simmons R.A. (2018). Mice exposed to bisphenol A exhibit depressive-like behavior with neurotransmitter and neuroactive steroid dysfunction. Horm. Behav..

[22] Yao J., Wang J., Wu L., Lu H., Wang Z., Yu P., Xiao H., Gao R., Yu J. (2020). Perinatal exposure to bisphenol A causes a disturbance of neurotransmitter metabolic pathways in female mouse offspring: A focus on the tryptophan and dopamine pathways. Chemosphere.

[23] Ponzi D., Gioiosa L., Parmigiani S., Palanza P. (2020). Effects of Prenatal Exposure to a Low-Dose of Bisphenol A on Sex Differences in Emotional Behavior and Central Alpha(2)-Adrenergic Receptor Binding. Int. J. Mol. Sci..

[24] Tando S., Itoh K., Yaoi T., Ogi H., Goto S., Mori M., Fushiki S. (2014). Bisphenol A exposure disrupts the development of the locus coeruleus-noradrenergic system in mice. Neuropathology.

[25] Hatcher K.M., Willing J., Chiang C., Rattan S., Flaws J.A., Mahoney M.M. (2019). Exposure to di-(2-ethylhexyl) phthalate transgenerationally alters anxiety-like behavior and amygdala gene expression in adult male and female mice. Physiol. Behav..

[26] Yirun A., Ozkemahli G., Balci A., Erkekoglu P., Zeybek N.D., Yersal N., Kocer-Gumusel B. (2021). Neuroendocrine disruption by bisphenol A and/or di(2-ethylhexyl) phthalate after prenatal, early postnatal and lactational exposure. Environ. Sci. Pollut. Res. Int..

[27] Kaimal A., Hooversmith J.M., Cherry A.D., Garrity J.T., Al Mansi M.H., Martin N.M., Buechter H., Holmes P.V., MohanKumar P.S., MohanKumar S.M.J. (2023). Prenatal exposure to bisphenol A and/or diethylhexyl phthalate alters stress responses in rat offspring in a sex- and dose-dependent manner. Front. Toxicol..

[28] Environmental Protection Agency (2010). Bisphenol A Action Plan (CASRN 80-05-7).

[29] Almeida S., Raposo A., Almeida-González M., Carrascosa C. (2018). Bisphenol A: Food Exposure and Impact on Human Health. Compr. Rev. Food Sci. Food Saf..

[30] Lakind J.S., Naiman D.Q. (2008). Bisphenol A (BPA) daily intakes in the United States: Estimates from the 2003-2004 NHANES urinary BPA data. J. Expo. Sci. Environ. Epidemiol..

[31] Blystone C.R., Kissling G.E., Bishop J.B., Chapin R.E., Wolfe G.W., Foster P.M. (2010). Determination of the di-(2-ethylhexyl) phthalate NOAEL for reproductive development in the rat: Importance of the retention of extra animals to adulthood. Toxicol. Sci..

[32] Wittassek M., Koch H.M., Angerer J., Brüning T. (2011). Assessing exposure to phthalates—The human biomonitoring approach. Mol. Nutr. Food Res..

[33] Environmental Protection Agency (1987). Integrated Risk Information System (IRIS) Chemical Assessment Summary: Di(2-ethylhexyl)phthalate (DEHP); CASRN 117-81-7. https://iris.epa.gov/static/pdfs/0014_summary.pdf.

[34] Simone J., Bogue E.A., Bhatti D.L., Day L.E., Farr N.A., Grossman A.M., Holmes P.V. (2015). Ethinyl estradiol and levonorgestrel alter cognition and anxiety in rats concurrent with a decrease in tyrosine hydroxylase expression in the locus coeruleus and brain-derived neurotrophic factor expression in the hippocampus. Psychoneuroendocrinology.

[35] Palkovits M. (1973). Isolated removal of hypothalamic or other brain nuclei of the rat. Brain Res..

[36] Balasubramanian P., Subramanian M., Nunez J.L., Mohankumar S.M., Mohankumar P.S. (2014). Chronic estradiol treatment decreases brain derived neurotrophic factor (BDNF) expression and monoamine levels in the amygdala–implications for behavioral disorders. Behav. Brain Res..

[37] Belujon P., Grace A.A. (2017). Dopamine System Dysregulation in Major Depressive Disorders. Int. J. Neuropsychopharmacol..

[38] Malikowska-Racia N., Sałat K., Nowaczyk A., Fijałkowski Ł., Popik P. (2019). Dopamine D2/D3 receptor agonists attenuate PTSD-like symptoms in mice exposed to single prolonged stress. Neuropharmacology.

[39] Linnet J. (2020). The anticipatory dopamine response in addiction: A common neurobiological underpinning of gambling disorder and substance use disorder?. Prog. Neuropsychopharmacol. Biol. Psychiatry.

[40] Vaessen T., Hernaus D., Myin-Germeys I., van Amelsvoort T. (2015). The dopaminergic response to acute stress in health and psychopathology: A systematic review. Neurosci. Biobehav. Rev..

[41] Boyce P.J., Finlay J.M. (2005). Neonatal depletion of cortical dopamine: Effects on dopamine turnover and motor behavior in juvenile and adult rats. Brain Res. Dev. Brain Res..

[42] George T.P., Verrico C.D., Roth R.H. (1998). Effects of repeated nicotine pre-treatment on mesoprefrontal dopaminergic and behavioral responses to acute footshock stress. Brain Res..

[43] Dunn A.J., Berridge C.W. (1987). Corticotropin-releasing factor administration elicits a stress-like activation of cerebral catecholaminergic systems. Pharmacol. Biochem. Behav..

[44] Olguín H.J., Guzmán D.C., García E.H., Mejía G.B. (2016). The Role of Dopamine and Its Dysfunction as a Consequence of Oxidative Stress. Oxid. Med. Cell. Longev..

[45] Klein M.O., Battagello D.S., Cardoso A.R., Hauser D.N., Bittencourt J.C., Correa R.G. (2019). Dopamine: Functions, Signaling, and Association with Neurological Diseases. Cell. Mol. Neurobiol..

[46] Feenstra M.G., Kalsbeek A., van Galen H. (1992). Neonatal lesions of the ventral tegmental area affect monoaminergic responses to stress in the medial prefrontal cortex and other dopamine projection areas in adulthood. Brain Res..

[47] Kalsbeek A., Feenstra M.G., van Galen H., Uylings H.B. (1989). Monoamine and metabolite levels in the prefrontal cortex and the mesolimbic forebrain following neonatal lesions of the ventral tegmental area. Brain Res..

[48] Molina-Holgado E., Dewar K.M., Grondin L., van Gelder N.M., Reader T.A. (1993). Amino acid levels and gamma-aminobutyric acidA receptors in rat neostriatum, cortex, and thalamus after neonatal 6-hydroxydopamine lesion. J. Neurochem..

[49] Luu B.E., Green S.R., Childers C.L., Holahan M.R., Storey K.B. (2017). The roles of hippocampal microRNAs in response to acute postnatal exposure to di(2-ethylhexyl) phthalate in female and male rats. Neurotoxicology.

[50] Smith C.A., Farmer K., Lee H., Holahan M.R., Smith J.C. (2015). Altered Hippocampal Lipid Profile Following Acute Postnatal Exposure to Di(2-Ethylhexyl) Phthalate in Rats. Int. J. Environ. Res. Public Health.

[51] Heidbreder C.A., Weiss I., Domeney A., Pryce C., Homberg J., Hedou G., Feldon J., Moran M., Nelson P. (2000). Behavioral, neurochemical and endocrinological characterization of the early social isolation syndrome. Neuroscience.

[52] Sullivan R.M., Szechtman H. (1995). Asymmetrical influence of mesocortical dopamine depletion on stress ulcer development and subcortical dopamine systems in rats: Implications for psychopathology. Neuroscience.

[53] Abercrombie E.D., Keller R.W., Zigmond M.J. (1988). Characterization of hippocampal norepinephrine release as measured by microdialysis perfusion: Pharmacological and behavioral studies. Neuroscience.

[54] Hansen N. (2017). The Longevity of Hippocampus-Dependent Memory Is Orchestrated by the Locus Coeruleus-Noradrenergic System. Neural Plast..

[55] Lovick T.A., Zangrossi H. (2021). Effect of Estrous Cycle on Behavior of Females in Rodent Tests of Anxiety. Front. Psychiatry.

[56] Wise L.M., Hernandez-Saavedra D., Boas S.M., Pan Y.X., Juraska J.M. (2019). Perinatal High-Fat Diet and Bisphenol A: Effects on Behavior and Gene Expression in the Medial Prefrontal Cortex. Dev. Neurosci..

[57] Nadeem A., Al-Harbi N.O., Ahmad S.F., Alhazzani K., Attia S.M., Alsanea S., Alhoshani A., Mahmood H.M., Alfardan A.S., Bakheet S.A. (2021). Exposure to the plasticizer, Di-(2-ethylhexyl) phthalate during juvenile period exacerbates autism-like behavior in adult BTBR T + tf/J mice due to DNA hypomethylation and enhanced inflammation in brain and systemic immune cells. Prog. Neuropsychopharmacol. Biol. Psychiatry.

[58] Almey A., Milner T.A., Brake W.G. (2015). Estrogen receptors in the central nervous system and their implication for dopamine-dependent cognition in females. Horm. Behav..

[59] Gillies G.E., McArthur S. (2010). Estrogen actions in the brain and the basis for differential action in men and women: A case for sex-specific medicines. Pharmacol. Rev..

[60] Kuiper G.G., Lemmen J.G., Carlsson B., Corton J.C., Safe S.H., Van Der Saag P.T., Van Der Burg B., Gustafsson J.Å. (1998). Interaction of estrogenic chemicals and phytoestrogens with estrogen receptor beta. Endocrinology.

[61] Perez P.A., Toledo J., Sosa L.d.V., Peinetti N., Torres A.I., De Paul A.L., Gutiérrez S. (2020). The phthalate DEHP modulates the estrogen receptors alpha and beta increasing lactotroph cell population in female pituitary glands. Chemosphere.

[62] Santoro A., Mele E., Marino M., Viggiano A., Nori S.L., Meccariello R. (2021). The Complex Interplay between Endocannabinoid System and the Estrogen System in Central Nervous System and Periphery. Int. J. Mol. Sci..

[63] Ernst J., Grabiec U., Falk K., Dehghani F., Schaedlich K. (2020). The endocrine disruptor DEHP and the ECS: Analysis of a possible crosstalk. Endocr. Connect..

[64] Salehi A., Loganathan N., Belsham D.D. (2019). Bisphenol A induces Pomc gene expression through neuroinflammatory and PPARgamma nuclear receptor-mediated mechanisms in POMC-expressing hypothalamic neuronal models. Mol. Cell. Endocrinol..

[65] Wojtowicz A.K., Sitarz-Glownia A.M., Wnuk A., Kajta M., Szychowski K.A. (2023). Involvement of the peroxisome proliferator-activated receptor gamma (Ppargamma) and matrix metalloproteinases-2 and -9 (Mmp-2 and -9) in the mechanism of action of di(2-ethylhexyl)phthalate (DEHP) in cultured mouse brain astrocytes and neurons. Toxicol. Vitr..

[66] Nishizawa H., Morita M., Sugimoto M., Imanishi S., Manabe N. (2005). Effects of in utero exposure to bisphenol A on mRNA expression of arylhydrocarbon and retinoid receptors in murine embryos. J. Reprod. Dev..

[67] Wojtowicz A.K., Sitarz-Glownia A.M., Szczesna M., Szychowski K.A. (2019). The Action of Di-(2-Ethylhexyl) Phthalate (DEHP) in Mouse Cerebral Cells Involves an Impairment in Aryl Hydrocarbon Receptor (AhR) Signaling. Neurotox. Res..

